# Physico-Chemically Distinct Nanomaterials Synthesized from Derivates of a Poly(Anhydride) Diversify the Spectrum of Loadable Antibiotics

**DOI:** 10.3390/nano10030486

**Published:** 2020-03-08

**Authors:** Amalia Mira, Carlos Sainz-Urruela, Helena Codina, Stuart I. Jenkins, Juan Carlos Rodriguez-Diaz, Ricardo Mallavia, Alberto Falco

**Affiliations:** 1 Institute of Research, Development and Innovation in Biotechnology of Elche (IDiBE), Miguel Hernández University (UMH), 03202 Elche, Alicante, Spain; a.mira@umh.es (A.M.); carlos.sainz@goumh.umh.es (C.S.-U.); helena.codina@goumh.umh.es (H.C.); 2 Neural Tissue Engineering group: Keele (NTEK), School of Medicine, Keele University, Keele ST5 5BG, Staffordshire, UK; s.i.jenkins@keele.ac.uk; 3 Microbiology Section, University General Hospital of Alicante, Alicante Institute for Health and Biomedical Research (ISABIAL-FISABIO Foundation), Alicante 03010, Spain; rodriguez_juadia@gva.es

**Keywords:** biomaterials, polymers, PMVE/MA, electrospinning, nanofibers, nanoparticles, nanoencapsulation, antibiotics

## Abstract

Recent advances in the field of nanotechnology such as nanoencapsulation offer new biomedical applications, potentially increasing the scope and efficacy of therapeutic drug delivery. In addition, the discovery and development of novel biocompatible polymers increases the versatility of these encapsulating nanostructures, enabling chemical properties of the cargo and vehicle to be adapted to specific physiological requirements. Here, we evaluate the capacity of various polymeric nanostructures to encapsulate various antibiotics of different classes, with differing chemical structure. Polymers were sourced from two separate derivatives of poly(methyl vinyl ether-*alt*-maleic anhydride) (PMVE/MA): an acid (PMVE/MA-Ac) and a monoethyl ester (PMVE/MA-Es). Nanoencapsulation of antibiotics was attempted through electrospinning, and nanoparticle synthesis through solvent displacement, for both polymers. Solvent incompatibilities prevented the nanoencapsulation of amikacin, neomycin and ciprofloxacin in PMVE/MA-Es nanofibers. However, all compounds were successfully loaded into PMVE/MA-Es nanoparticles. Encapsulation efficiencies in nanofibers reached approximately 100% in all compatible systems; however, efficiencies varied substantially in nanoparticles systems, depending on the tested compound (14%–69%). Finally, it was confirmed that both these encapsulation processes did not alter the antimicrobial activity of any tested antibiotic against *Staphylococcus aureus* and *Escherichia coli*, supporting the viability of these approaches for nanoscale delivery of antibiotics.

## 1. Introduction

So far, antibiotics are the most reliable weapon against infectious diseases of bacterial origin. However, the dramatic diminishment in the rate of discovery of new antibiotics limits our response to pathogens resistant to conventional antibiotics and to new emerging diseases. In response, the scientific community is developing alternative strategies to increase the effectiveness, and/or to overcome the limitations, of existing antibiotics [1,2]. In this context, recent advances in the field of nanotechnology offer new tools such as nanoencapsulation: the loading of pharmaceutical agents within nanomaterials [3,4]. 

From a commercial and clinical point of view, nanoencapsulation can protect pharmaceutical products, extending both shelf-life and biological half-life (e.g., in circulation). In addition, with the corresponding modifications, these nanostructured systems can facilitate targeted drug delivery and/or specific controlled-release kinetics, thereby increasing the effectiveness of the treatment and, thus, reducing necessary dosage and side effects [3,4]. 

In line with this, the continuing development of novel biocompatible polymers contributes to the potential versatility of these nanostructures, by enabling the delivery of compounds with solubility limitations. In addition, manipulation of synthesis or layering protocols can generate nanostructures with properties tailored to highly specific applications, for example injectable nanoparticles, or nanofibers for wound dressings [5,6,7,8].

Examples of such highly versatile materials include derivates of poly(methyl vinyl ether-*alt*-maleic anhydride) (PMVE/MA), an alternating copolymer of methyl-ether-vinyl and maleic anhydride. This material is marketed by Ashland Inc. as Gantrez^®^ and presents suitable properties for biomedical applications (low toxicity, high biocompatibility, high mucoadhesivity and low cost). In particular, it is reported that PMVE/MA can be structured as loadable nanoparticles [9] and its derivates poly(methyl vinyl ether-alt-maleic acid) (PMVE/MA-Ac) and poly(methyl vinyl ether-alt-maleic monoethyl ester) (PMVE/MA-Es) as nanofibers [10,11]. In addition, these polymers have shown utility as matrix elements, allowing the combination with other materials to generate novel mixtures of chemical properties in the final nanostructures [11,12,13], such as with homemade fluorescent cationic fluorene-based polyelectrolytes [11,14,15,16].

In this work, both nanoparticles and nanofibers were synthesized using these polymers, and their relative capacities for encapsulation were compared. There are several possible procedures for the synthesis of each of these nanomaterials. For instance, solvent displacement, emulsion solvent diffusion, interfacial deposition and nanoprecipitation synthesis for nanoparticles [17] and electrospinning, self-assembly, phase separation and template synthesis for nanofibers [18]. The two nanostructuring methodologies utilized here (solvent displacement and electrospinning, for particle and fiber synthesis, respectively), although differing markedly in terms of the processes involved, were selected because of the high degree of uniformity in their products, as well as being scalable processes for industrial production. The range of compounds tested as cargo encompassed four antibiotics of three different classes, differing in their molecular weights, structures and modes of action, i.e., two aminoglycosides (amikacin and neomycin), a cephalosporin (cefotaxime) and a quinolone (ciprofloxacin). Finally, the antimicrobial activity of the encapsulated compounds, as well as their structural stability, were also assessed, to confirm that nanoencapsulation was not an impairment to their antimicrobial potency.

## 2. Materials and Methods 

### 2.1. Materials

**Figure 1:**
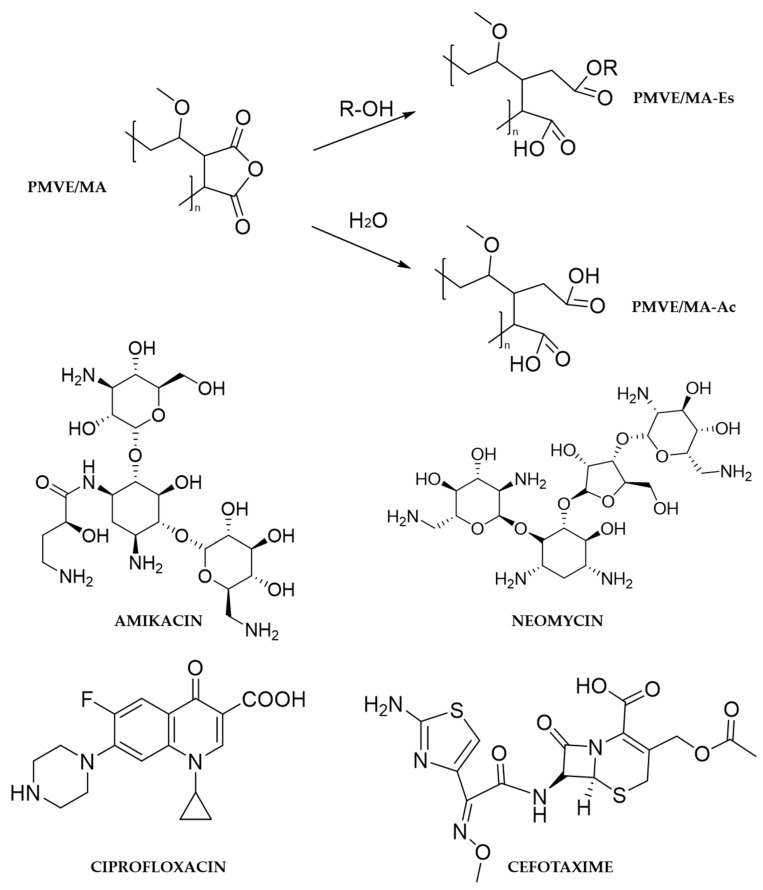
Structures of the principal compounds used. All structures were drawn using ChemBioDraw Ultra v14.0 (CambridgeSoft, Cambridge, MA, USA).

The polymers PMVE/MA-Es (CAS number: 25087-06-3; MW: 130 kg/mol), provided as 50% *w*/*w* solution in ethanol, and PMVE/MA-Ac (CAS number: 25153-40-6; MW: 216 kg/mol), in powder format, were purchased from Sigma-Aldrich (St. Louis, MO, USA). Amikacin (Mw: 585.6 g/mol; 250 mg/mL) and cefotaxime (MW: 455.5 g/mol; powder) were acquired from Laboratorios Normon (Tres Cantos, Spain). Neomycin (MW: 614.6 g/mol; 10 mg/mL) was obtained from Sigma-Aldrich and ciprofloxacin (MW: 331.3 g/mol; 2 mg/mL) from Genéricos Españoles laboratorios S.A. (Las Rozas, Spain). The chemical structure inputs of all these compounds are shown in Figure 1.

Phosphate buffered saline (PBS; pH 7.4), Mueller-Hinton broth (MHB; powder format), methanol, acetone, phosphoric acid and trifluoroacetic acid with HPLC grade purity, and dimethylsulfoxide (DMSO) and ethanol with purity >95%, were all from Sigma-Aldrich. The water used was double distilled and deionized (DDW) from a Milli-Q Synthesis A10 system (Millipore, Madrid, Spain).

### 2.2. Preparation of Nanofibers by Electrospinning

From our previous studies, either 20% *w*/*w* PMVE/MA-Ac in H_2_O or 25% *w*/*w* PMVE/MA-Es in ethanol were selected as optimal polymeric solutions for the creation of electrospun nanofibers [10,11]. Antibiotics were added to the polymeric solutions to reach a final concentration of 1% *w*/*w* with respect to both PMVE/MA-Ac and PMVE/MA-Es. All solutions were stirred for 1 h and then bath-sonicated for 10 min prior to electrospinning.

As for the electrospinning system, the polymer solutions inside a 2-mL Discardit II syringe (Becton Dickinson, Franklin Lakes, NJ, USA) were pumped through a 20-Gauge blunt-end stainless steel hypodermic needle 316 (Sigma-Aldrich) at a constant flow rate by using a KDS 100 infusion pump (KD Scientific, Holliston, MA, USA). A Series FC high voltage supplier (Glassman High Voltage Inc., Whitehouse Station, NJ, USA) was responsible for the generation of the electrostatic field, which focused the jet onto a collector, made of aluminum foil and located in front of the syringe tip (vertical orientation). Any surface intended to be covered with a mat of electrospun nanofibers would be placed on the collector, for instance common glass slides for fluorescence/optical microscopy analysis or copper grids (diameter 3 mm) for transmission electron microscopy (TEM; Electron Microscopy Sciences, Hatfield, PA, USA). Operational parameters: 15 kV, flow rate 0.5 mL/h, needle-collector distance 15 cm, room temperature (RT) and relative humidity 40%–60%. Finally, in order to evaporate solvent excess, the obtained mats were kept in a fume hood overnight. Synthesized nanofibers were then stored protected from light at RT and dry atmosphere until used.

### 2.3. Preparation of Nanoparticles by Solvent Displacement

The PMVE/MA-Es nanoparticles were created using the solvent displacement procedure previously described by Arbós et al. (2002) [19] with some modifications. Briefly, different amounts of PMVE/MA-Es (12.5–200 mg) were dissolved in up to 5 g of ethanol by means of magnetic agitation for 10 min at RT. Subsequently, 2 µg of antibiotic in DDW were added (control nanoparticles were prepared only with DDW). After 10 min further magnetic stirring, gradual addition of DDW brought the final volume to 15 g. Then, the ethanol was evaporated under reduced pressure (BUCHI Rotavapor R-230, Flawil, Switzerland). Finally, obtained nanoparticles were purified twice by centrifugation (Sigma 3K30, Sigma Instruments, Osterode, Germany) at 20,000 g for 20 min at 4 °C. The pellet was then resuspended in up to 10 g of DDW and thus the final concentrations were 0.125%–2% *w*/*w* (or 1.25–20 mg/g) of PMVE/MA-Es and 200 ng/g of antibiotic. The supernatants were further centrifuged at 30,000 g for 30 min at 4 °C and collected again to quantify the amount of unloaded antibiotics. The purified nanoparticles and supernatants from all batches were stored at −20 °C until use.

### 2.4. Average Size and Zeta Potential Determination of Nanoparticles

Photon correlation spectroscopy (PCS), also known as dynamic light scattering (DLS), was used to determine the average hydrodynamic diameter (HDD) and polydispersity index (PDI) of each batch of nanoparticles (90 Plus Nanoparticle Size Analyzer; 35 mV red diode laser source, ʎ = 640 nm; Brookhaven Instruments Corporations, Holtsville, NY, USA). Each suspension sample was diluted with DDW to yield an appropriate scattering intensity of 100–400 kcps. All measurements were performed three times at 25 °C with angle detection fixed at 90° on 2 mL samples.

The zeta potential (ZP) of the synthesized nanoparticles was determined by electrophoretic laser Doppler anemometry with the 90 Plus Particle Size Analyzer (Brookhaven Instruments Corporation). Suspension samples were prepared as described before and analyzed in triplicate. All results are shown as the mean and standard deviation (s.d.) of the values obtained from three different batches.

### 2.5. Microscopy

For optical microscopy initial screenings, the nanofibers were electrospun on microscope slides (Deltalab, Barcelona, Spain) and observed by means of a Mycrosystems DMI3000B inverted fluorescence microscope equipped with an EL6000 compact light source and a DFC 3000G digital camera, all from Leica (Bensheim, Germany). All images were taken with a 63× objective in phase contrast and image processing performed manually using the software Leica Application SuiteAF6000 Module Systems.

Detailed observations of selected nanofiber samples were carried out by scanning electron microscopy (SEM), without metal coating, in a JSM-6360 LV device (Jeol, Tokyo, Japan). For size analysis, diameter measurements were performed on 100 nanofibers (minimum three micrographs) per nanofiber synthesis condition using ImageJ software (National Institutes of Health, NIH, Bethesda, MD, USA).

Transmission electron microscopy (TEM) was used to confirm the size and describe the morphology of the synthesized nanoparticles (Jeol 1011 apparatus, at 120 kV). Samples were placed onto Formvar/carbon 300-Mesh, copper grids (Electron Microscopy Science, Hatfield, PA, USA) and then incubated with citrate lead solution (0.03% p/v) to generate contrast in images. Diameter measurements were performed on 50 nanoparticles (minimum three micrographs) per nanoparticle synthesis condition using ImageJ software (National Institutes of Health).

### 2.6. HPLC Analysis

The quantitation of antibiotics was performed by HPLC in order to determine their encapsulation efficiency (EE, percentage of encapsulated compound relative to the theoretically maximum one) in both nanostructures. For nanoparticles, non-loaded compound, i.e., free in supernatants obtained during synthesis prior to purification, was quantitated then subtracted from the total added antibiotics to determine the amount of loaded compound. Nanoparticle supernatants or 0.1% *w*/*w* nanofiber solutions in DDW were filtered through a 0.4 µm PTFE membrane (Chmlab group, Barcelona, Spain) prior to volume injections (10 µL for amikacin and neomycin, 20 µL for ciprofloxacin and 5 µL for cefotaxime). 

A Merck-Hitachi D-7000 HPLC system (Hitachi Instruments, Tokyo, Japan) equipped with an Alltech 3300 evaporative light scattering detector (ELSD; Alltech Associates Inc., Lokeren, Belgium) was used to analyze amikacin and neomycin. Cefotaxime and ciprofloxacin were analyzed by means of an Agilent LC 214 1100 series HPLC system controlled by ChemStation software and equipped with a G1311A quaternary pump, a G1329A ALS automated sample injector, a G1316A thermostat column compartment and a G1316A diode array detector (Agilent Technologies, Inc., Palo Alto, CA, USA).

All methods used were isocratic and their particularities for each antibiotic were as follows. For amikacin and neomycin, the mobile phase was acetone:DDW with 0.15% *v*/*v* TFA at a 1:1 ratio, a flow rate at 1.0 mL/min. ELSD conditions were nitrogen pressure at 3.5 bar and temperature at 45 °C. For cefotaxime, the mobile phase was methanol:DDW at 30:70 *v*/*v* (adjusted to pH 4 with acetic acid), a flow rate at 0.8 mL/min and detection at 235 nm. For ciprofloxacin, the mobile phase was 0.25 M phosphoric acid:acetonitrile at 75:25 *v*/*v*, a flow rate at 0.8 mL/min and detection at 280 nm. Standard curves for each antibiotic were generated using the same concentration range (6.25–300 µg/mL). Within this range, a linear correlation was found between concentration and detected peak area with regression coefficient values (R^2^) greater than 0.99 (Figure S1).

### 2.7. Antibacterial Assays

The antimicrobial activity of the experimental formulations was tested on antibiotic-sensitive strains of Gram-positive *Staphylococcus aureus* (CECT 59) and Gram-negative *Escherichia coli* (CECT 515) obtained from the Spanish Type Culture Collection (Colección Española de Cultivos Tipo, CECT, Universitat de Valencia, Spain). Prior to each assay, a colony of bacteria previously grown in MHB-agar plates was isolated and incubated in MHB for 12 h at 37 °C to prepare bacteria inocula.

Minimal inhibitory concentration (MIC) was determined by the two-fold broth microdilution method according to the Clinical and Laboratory Standards Institute (CLSI) guidelines [20], with some modifications. Briefly, two-fold dilutions in MHB of the experimental formulations and the control antibiotics at twice the final concentration were prepared and 50 µL/well were dispensed in each column (1 column per concentration) of round-bottom 96-well polystyrene plates (Deltalab S.L., Rubí, Spain). Then, bacteria suspension, after adjustment to 0.5 McFarland and then further diluted by 1:100 in MHB, was added to all wells (50 µL/well), except for sterile controls. Plates were then incubated for 24 h at 37 °C and finally MIC was defined as the lowest concentration of antibiotic that visibly inhibited the growth of the bacterium being investigated. All assays were performed in triplicate and results are shown as the mean and s.d.

### 2.8. Data Analysis and Graphics

Data were analyzed and graphs produced using GraphPad Prism v6 and Microsoft Excel software. Statistical analysis was performed with GraphPad Prism v6. 

## 3. Results

### 3.1. Electrospinnability of Formulations and Characterization of Obtained Nanofibers

In our previous works [10,11], the optimal conditions for the electrospinning of the polymer solutions for both of these PMVE/MA derivates were determined, and hence taken as starting points for the present study (briefly, 15 kV, flow rate of 0.5 mL/h, needle-collector distance of 15 cm and polymer concentrations of 20% *w*/*w* PMVE/MA-Ac in H_2_O or 25% *w*/*w* PMVE/MA-Es in ethanol). In this study, such conditions proved suitable for generating morphologically uniform nanofibers when initial polymeric solutions were homogenous. However, amikacin, neomycin and ciprofloxacin were found to be insoluble in ethanol, and therefore insoluble in PMVE/MA-Es solutions. Therefore, nanofibers were obtained from PMVE/MA-Ac solutions in combination with all antibiotics, but PMVE/MA-Es solution was only used with cefotaxime.

**Figure 2:**
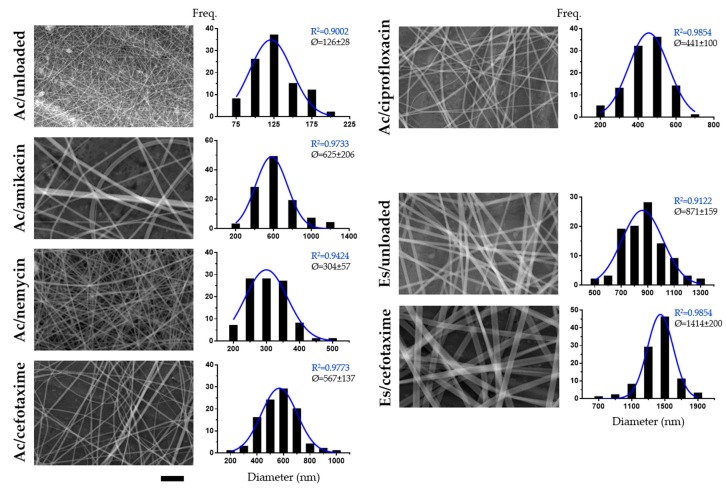
SEM analysis of antibiotic-loaded electrospun nanofibers. Representative SEM micrographs and corresponding diameter frequency histograms for PMVE/MA-Ac, PMVE/MA-Ac/amikacin, PMVE/MA-Ac/neomycin, PMVE/MA-Ac/cefotaxime, PMVE/MA-Ac/ciprofloxacin, PMVE/MA-Es and PMVE/MA-Es/cefotaxime. Histogram data were obtained from multiple micrographs (100 individual measurements). Best-fit adjustments (and their R^2^) to a Gaussian distribution are indicated in blue. Average diameter (Ø) ± s.d. is also stated. Scale bar: 5 µm.

From both optical microscopy (data not shown) and SEM images (Figure 2), the morphology of all nanofibers was observed to be uniform (no shape anomalies), continuous (appropriate length, no breaks) and with a smooth surface appearance (no visible pores).

As SEM offers higher resolution and contrast imaging, as well as broader depth of field, these images were used for size analysis (Figure 2). All nanofiber types showed average diameter values < 1000 nm, except PMVE/MA-Es/cefotaxime (1414 ± 200 nm). PMVE/MA-Ac nanofibers with no encapsulated compound were the narrowest in diameter (126 ± 28 nm), which contrasts starkly with non-loaded PMVE/MA-Es fibers (871 ± 159 nm). The encapsulation of compounds increased the diameter of both PMVE/MA-Es and PMVE/MA-Ac nanofibers by a minimum of 1.6-fold (PMVE/MA-Es/cefotaxime) and a maximum of 5.0-fold (PMVE/MA-Ac/amikacin). Among PMVE/MA-Ac nanofibers, the smallest diameter increase was found when loading neomycin (2.4-fold), then ciprofloxacin (3.5-fold), with cefotaxime increasing the most (4.5-fold). In terms of size variability, PMVE/MA-Ac/amikacin, PMVE/MA-Ac/cefotaxime, PMVE/MA-Ac/ciprofloxacin and PMVE/MA-Es/cefotaxime nanofibers most closely fitted a Gaussian distribution (R^2^ > 0.95), although all formulations showed R^2^ values higher than 0.9. Of note, EEs reached maximum levels (≥97%) with relatively low s.d. (±2%) in all cases.

### 3.2. Optimization of the Preparation of PMVE/MA-Es Nanoparticles and Their Characterization

The low solubility in ethanol of most of the antibiotics tested, and the low solubility in DDW of PMVE/MA-Es, severely limited their compatibility for encapsulation in electrospun PMVE/MA-Es nanofibers. In contrast, encapsulation of these antibiotics in PMVE/MA-Es nanoparticles would appear feasible, since their preparation by the solvent displacement method requires, initially, both much lower polymer and ethanol concentrations (32.33% *w*/*w*). This method continues with the selective evaporation of ethanol to reach final nanoparticle suspensions in just DDW.

Preliminarily, the process of preparation of PMVE/MA-Es nanoparticles was optimized. For this purpose, several concentrations of PMVE/MA-Es were tested, ranging from 0.125% to 2% *w*/*w* (thus, 1.25–20 mg/g) in the final formulation. The physico-chemical parameters analyzed for these nanoparticles are summarized in Table 1. Overall, within the concentration range tested, greater polymer concentration was associated with larger nanoparticle diameter. No associations were noted between polymer concentration and either PDI or ZP parameters. PDI values ranged 0.06-0.20, indicating suspensions with high quality dispersion in general. In contrast, ZP values were more variable (from −5 to −35 mV), but only nanoparticles made of 10 and 20 mg/g PMVE/MA-Es displayed values yielding fairly good physical stability (≥30 mV approximately). Given these results, nanoparticles made of 10 mg/g PMVE/MA-Es were selected for further studies.

**Figure 3:**
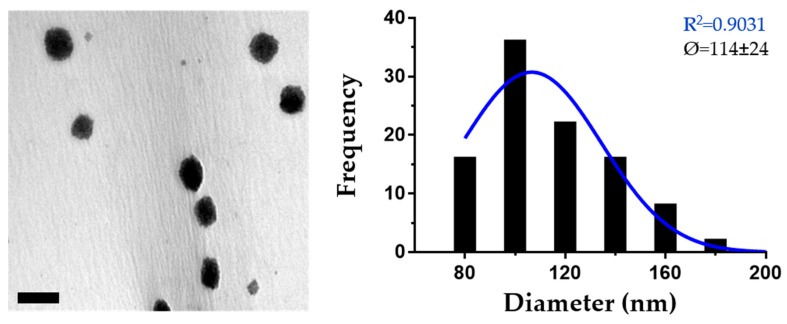
TEM analysis of optimized PMVE/MA-Es nanoparticles. Representative TEM image and diameter frequency histogram of PMVE/MA-Es nanoparticles synthesized by the solvent displacement method. The histogram was generated from data obtained from multiple images, until reaching 50 individual measurements. The best-fit adjustment (and R^2^) to a Gaussian distribution is indicated in blue. Average diameter (Ø) and s.d. are also included in the inset. Scale bar: 200 nm.

Such nanoparticle formulations were then analyzed by TEM (Figure 3), which revealed consistent spherical shapes and, in contrast to PCS results, not only a lower average size (114 ± 24 nm), but also a less homogeneous population, as can be observed from the frequency histogram of their diameters.

### 3.3. Characterization of PMVE/MA-Es Nanoparticles Loaded with Antibiotics

By following the methodology described above, antibiotic-loaded PMVE/MA-Es nanoparticles were prepared containing 10 mg/g PMVE/MA-Es and 200 ng/g antibiotic (if 100% EE). The physico-chemical characteristics and EE displayed by these formulations are summarized in Table 2. Control (non-loaded) nanoparticles showed similar physico-chemical values as observed before (previous section), confirming the high reproducibility of the technique. As for loaded nanoparticles, there were significant compound-dependent variations in these parameters. For instance, both amikacin- and ciprofloxacin-loading was associated with greater particle size, but only the latter showed a notably change in ZP value (−16 ± 3 mV). Nanoparticles loaded with cefotaxime showed lower diameter (156 ± 6 nm) in comparison to control, and only neomycin-loaded particles showed no substantial differences compared to controls. Regarding their EE, only amikacin showed relatively low values (14% ± 4%), with the other antibiotics ranging from 40% to 69%.

### 3.4. Analysis of the Antibacterial Activity

The antibacterial activity of encapsulated antibiotics was assessed against Gram-positive (*S. aureus*) and Gram-negative (*E. coli*) bacteria by determining their MIC values and comparing to those obtained for the free drug. The MIC values displayed by the encapsulated and free antibiotics were not significantly different (data not shown). As free compounds, MIC values for amikacin, neomycin, ciprofloxacin and cefotaxime were 1.25, 0.31, 2.5 and 0.16 µg/mL against *S. aureus* and 2.5, 1.25, 0.06 and 0.004 µg/mL against *E. coli*, respectively.

## 4. Discussion

The present work provides evidence of the versatility of polymers for generating nanostructures adapted to the solubility properties of the compounds of interest, for successful encapsulation of these compounds. Our previous studies provided the first demonstration of electrospinning procedures optimized for creating nanofibers of the two PMVE/MA derivates used here [10,11]. These were used to encapsulate 5-aminolevulinic acid (5-ALA) into nanofibers made of each polymer [11], and a combination of salicylic acid, methyl salicylate and capsaicin into PMVE/MA-Es nanofibers [10]. Here, we demonstrated the encapsulation of four structurally different molecules (with a common application: antibiotics) into electrospun nanofibers of either PMVE/MA-Ac or PMVE/MA-Es. All the antibiotics tested were soluble in water, and thus encapsulate into PMVE/MA-Ac nanofibers, but only cefotaxime was also soluble in ethanol, and consequently the only one loadable into PMVE/MA-Es nanofibers. In this regard, as many drugs are hydrophobic, the description of electrospinnable polymers with properties compatible for encapsulating hydrophobic compounds, is of great importance in order to incorporate such molecules into electrospun nanofibers while maintaining their biological functionality. This issue has been revisited by several authors recently [21,22]. Furthermore, these polymers might also work as a shell phase in both co-axial and layer-by-layer electrospinning in order to either manipulate the delivery dynamics of high hydrophilic molecules to the particular requirements or to adapt the delivery system to the needs of the biological context [23,24].

Although the morphology of the nanofibers was not affected by the encapsulation of molecules, their diameter did undergo considerable changes in comparison to non-loaded fibers. The extent of these increases in diameter was related to the compound used (1.6-fold for PMVE/MA-Es/cefotaxime nanofibers and 2.4–5.0-fold for loaded PMVE/MA-Ac nanofibers). These increases were larger than expected, as only 1% *w*/*w* antibiotic was used in all cases and loading percentages for relatively similar compounds are usually much higher with no, or even reduced, diameter [10,25,26]. In general, within moderate encapsulation levels, the diameter of electrospun nanofibers commonly increases with viscosity and decreases with conductivity [25,26,27,28,29], and since the compounds used here are not salts or polyelectrolytes, they might be increasing the viscosity of the electrospinnable solution [25,26]. In any case, the current study employs the same synthesis parameters for all formulations in order to reliably determine any possible morphological or size changes between the different antibiotics incorporated, and if it is desirable to strictly limit any increase in size, it might be achievable by optimizing such parameters for each particular formulation, as shown in our previous studies [10,11].

This work has shown the flexibility of nanostructuration of some polymers and offers alternative encapsulating nanomaterials for compounds whose solubility limitations hinder their biological/therapeutic applications. Although amikacin, neomycin and ciprofloxacin could not be loaded into PMVE/MA-Es nanofibers by electrospinning, the solubility of all integrating molecules with water:ethanol solutions allowed their encapsulation into PMVE/MA-Es nanoparticles, synthesized by the solvent displacement method. This methodology has been widely reported to create PMVE/MA nanoparticles for drug delivery purposes because of the interesting mucoadhesive properties of this polymer [19,30]; however, to our knowledge, there are no publications describing nanoparticles made of PMVE/MA-Es apart from the patent with reference number US20140161892A1 [31].

The optimized methodology for encapsulating particles (10 mg/g PMVE/MA-Es) produced polymeric nanoparticles with size (200 nm), PDI (0.2) and ZP (−35 mV). Briefly, at the concentrations tested, the greater the polymer concentration used, the larger the nanoparticle diameter produced, while formulations with smaller nanoparticles showed less adequate PDI and ZP values than the selected one. The PDI, which ranges from 0 to 1, reflects the width of the particle size distribution and values lower than 0.3 are commonly considered as optimum [32]. Regarding the ZP, which reflects the level of surface electrostatic potential, values of approximately −30 mV are associated with physical stability and are considered at the limit for colloidal organizations (theoretically, about −60 mV minimum is considered optimal) [33,34]. Comparing to these results, loaded nanoparticles did not differ much in the PDI, ZP and size values obtained, which is in accordance with some studies [35,36,37,38,39].

In contrast, the size of PMVE/MA-Es nanoparticles did change notably (increased by about 25%) for some antibiotics, although not as dramatically as in loaded PMVE/MA-Ac nanofibers. Interestingly, the greatest size increases were found when encapsulating amikacin into both types of nanostructures; however, no further correlations could be established for the remaining antibiotics. These size changes do not appear to be related to their MWs, nor to their EEs. For instance, despite the two aminoglycosides tested, amikacin and neomycin, presenting similar MWs (585.6 and 614.6 g/mol, respectively) and chemical structures (see Figure 1), the size change of their encapsulating nanostructures (625 vs. 304 nm in nanofibers and 253 vs. 209 nm in nanoparticles) and their EEs (14% vs. 40%) are very different. In this regard, the scarcity of nanoencapsulation studies for amikacin, neomycin cefotaxime and, to a lesser extent, ciprofloxacin [40,41,42,43], hampers any meaningful assessment or comparison.

Nevertheless, all tested antibiotics, when encapsulated into either nanomaterial, showed similar antibacterial activity as their free forms, indicating that none of the synthesis methodologies used substantially modified their chemical structures. This observation encourages the performance of follow-up experiments focused on assessing any potential advantages conferred by the described encapsulating nanomaterials in terms of controlled release, targeted delivery or other therapeutic impact. Any such advantages may help to overcome the drawbacks restricting the clinical use of some therapeutics (such as toxicity, poor penetration of biological barriers and short circulating half-life) [44].

## 5. Conclusions

The compatibility in solubility of both the polymer source and the compound of interest is a critical factor limiting the production of loaded polymeric electrospun nanofibers. However, it has been demonstrated here that the high versatility with which some polymers can be assembled into nanomaterials, in this work PMVE/MA-Ac and PMVE/MA-Es, offers novel encapsulating strategies to overcome such constraints. In this context, it has also been shown that the encapsulation efficiency, nanostructuration settings and/or the morphology/size of the final nanostructures are highly dependent upon the chemical properties, rather than the molecular weight, of the compounds to be encapsulated. In contrast, it is confirmed that none of the nanostructuring procedures employed alters the activity of a set molecules with different chemical structures. 

## Figures and Tables

**Table 1:** Physico-chemical characteristics of nanoparticles made with different PMVE/MA-Es concentrations.

Conc. (mg/g)	HDD (nm)	PDI (a.u.)	ZP (mV)
**1.25**	84 ± 5	0.11 ± 0.04	−26 ± 10
**2.5**	139 ± 26	0.19 ± 0.03	−5 ± 4
**5**	106 ± 4	0.10 ± 0.04	−5 ± 6
**10**	202 ± 11	0.20 ± 0.06	−35 ± 9
**20**	230 ± 1	0.06 ± 0.01	−32 ± 1

Conc., concentration in the final formulation. a.u., arbitrary units. Results shown as the mean ± s.d. (*n* = 3).

**Table 2:** Physico-chemical characteristics and encapsulation efficiency (EE) of PMVE/MA-Es nanoparticles loaded with antibiotics.

Antibiotic	HDD (nm)	PDI (a.u.)	ZP (mV)	EE (%)
**None**	195 ± 7	0.15 ± 0.04	−38 ± 13	-
**Amikacin**	253 ± 5	0.17 ± 0.07	−39 ± 2	14 ± 4
**Neomycin**	209 ± 4	0.20 ± 0.07	−29 ± 7	40 ± 3
**Cefotaxime**	156 ± 6	0.29 ± 0.03	−31 ± 8	69 ± 7
**Ciprofloxacin**	256 ± 8	0.17 ± 0.01	−16 ± 3	59 ± 8

a.u., arbitrary units. Results shown as the mean and s.d. (*n* = 3).

## Supplementary Materials

The following are available online at https://www.mdpi.com/2079-4991/10/3/486/s1, Figure S1: Representative HPLC chromatograms and calibration curves of tested antibiotics.
