# Supplementary material for: Physico-Chemically Distinct Nanomaterials Synthesized from Derivates of a Poly(Anhydride) Diversify the Spectrum of Loadable Antibiotics
Source: Nanomaterials (Basel). 2020 Mar 8;10(3):486. doi: 10.3390/nano10030486 (PMC7153258; doi:10.3390/nano10030486)
Supplement: Supplementary file 1 [file nanomaterials-10-00486-s001.pdf]

## Supplementary figure 1

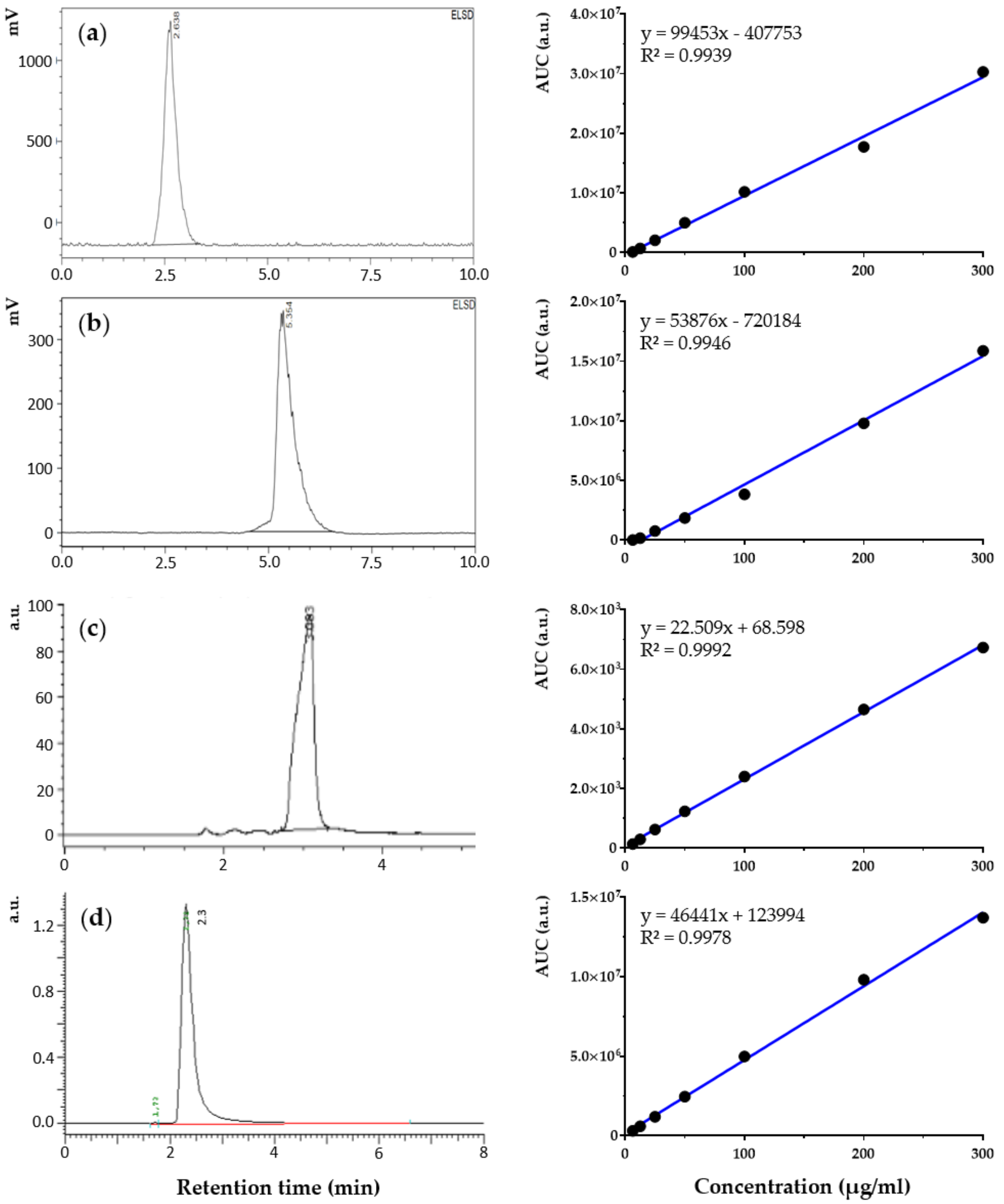


**Figure S1.** Representative HPLC chromatograms (left panels) and calibration curves (right panels) of (**a**) amikacin, (**b**) neomycin, (**c**) cefotaxime and (**d**) ciprofloxacin. Linear regressions are drawn in blue and the corresponding formulas and $R^2$ included in the insets. a.u., arbitrary units.
